# Acute Effects of the Wim Hof Breathing Method on Repeated Sprint Ability: A Pilot Study

**DOI:** 10.3389/fspor.2021.700757

**Published:** 2021-08-25

**Authors:** Tom Citherlet, Fabienne Crettaz von Roten, Bengt Kayser, Kenny Guex

**Affiliations:** ^1^Institute of Sport Sciences, University of Lausanne, Lausanne, Switzerland; ^2^School of Health Sciences, University of Applied Sciences and Arts Western Switzerland, Lausanne, Switzerland; ^3^Swiss Athletics, Haus des Sports, Ittigen, Switzerland

**Keywords:** Wim Hof breathing method, hyperventilation, apnea, RAST, anaerobic performance

## Abstract

The Wim Hof breathing method (WHBM) combines periods of hyperventilation (HV) followed by voluntary breath-holds (BH) at low lung volume. It has been increasingly adopted by coaches and their athletes to improve performance, but there was no published research on its effects. We determined the feasibility of implementing a single WHBM session before repeated sprinting performance and evaluated any acute ergogenic effects. Fifteen amateur runners performed a single WHBM session prior to a Repeated Ability Sprint Test (RAST) in comparison to voluntary HV or spontaneous breathing (SB) (control) in a randomized cross-over design. Gas exchange, heart rate, and finger pulse oxygen saturation (SpO_2_) were monitored. Despite large physiological effects in the SpO_2_ and expired carbon dioxide (VCO_2_) levels of both HV and WHBM, no significant positive or negative condition effects were found on RAST peak power, average power, or fatigue index. Finger SpO_2_ dropped to 60 ± 12% at the end of the BHs. Upon the last HV in the WHBM and HV conditions, end-tidal CO_2_ partial pressure (PETCO_2_) values were 19 ± 3 and 17 ± 3 mmHg, indicative of respiratory alkalosis with estimated arterial pH increases of +0.171 and of +0.181, respectively. Upon completion of RAST, 8 min cumulated expired carbon dioxide volumes in the WHBM and HV were greater than in SB, suggesting lingering carbon dioxide stores depletion. These findings indicate that despite large physiological effects, a single WHBM session does not improve anaerobic performance in repeated sprinting exercise.

## Introduction

Wim Hof is a Dutch athlete, nicknamed “Iceman” for his ability to withstand freezing temperatures. He has accumulated 20 “world records” for feats such as standing in a container while covered with ice cubes for 2 h, climbing Mount Kilimanjaro in shorts, swimming 60 m underneath ice, and running a half marathon barefoot on snow and ice north of the Arctic Circle (Hof, 2020c). He attributes these feats to training with his Wim Hof Method (WHM). This is a combination of breathing exercises [Wim Hof breathing method (WHBM): periods of hyperventilation (HV) followed by voluntary breath-holds (BH) at low lung volume (Hof, 2020b)], repeated exposure to cold, and mental commitment (Hof, 2020e). The WHM allegedly provides benefits such as stress reduction, enhanced creativity, more focus and mental clarity, better sleep, improved cardiovascular health, and improved exercise performance (Hof, 2020b). The latter would include faster recovery from physical exertion, heightened focus and mental composure, and increased muscular endurance (Hof, 2020d).

Many athletes have adopted the WHM, such as the tennis player Novak Djokovic (Novak Djokovic on Instagram: @iceman_hof how did we do?…, 2021), the surfer Kelly Slater (Kelly Slater's Bizarre, Daredevil-Inspired Breathing Technique, 2021), the American football punter Steve Weatherford (Hof, 2020a), the rower Janneke van der Meulen (Hof, 2020d), the UFC fighter Alistair Overeem, and the big wave surfer Laird Hamilton (Hof, 2021).

While the WHM seems to present interesting benefits, there is virtually no published research on its effects on sport performance. However, studies have shown that HV, which is part of the WHBM, can improve anaerobic performance (Ziegler, 2002; Sakamoto et al., 2014; Jacob et al., 2015). HV induces hypocapnia and drives the reaction sequence $\;H^{+}+HCO_{3}^{-}↔\;H_{2}CO_{3\;}↔\;H_{2}O+CO_{2}$ more to the right, elevating blood pH (Saladin and Miller, 2004). This respiratory alkalosis may improve anaerobic performance by compensating exercise-induced metabolic acidosis (Jacob et al., 2008). The effects on the performance of HV, combined with BH, as done in the WHBM, have not been investigated yet.

By itself, BH drives the reaction sequence $H^{+}+HCO_{3}^{-}↔\;H_{2}CO_{3\;}↔\;H_{2}O+CO_{2}\;$ more to the left, inducing a respiratory acidosis (Pflanzer, 2004). Thus, in the WHBM, the BH-induced CO_2_ retention would counter the HV-induced respiratory alkalosis. BH also triggers the so-called “diving response” (Foster and Sheel, 2005), which includes bradycardia, peripherical vasoconstriction, increased blood pressure, and contraction of the spleen (Dujic et al., 2011). The latter releases ~100 ml of concentrated red cells into the circulation, which may influence performance, even though most investigations did not find improvement in performance following apneas (Du Bois et al., 2014; Sperlich et al., 2015; Yildiz, 2018).

Any effects of HV or the WHBM are expected to be short-lasting and most likely to occur in short duration anaerobic lactic type performance such as the Wingate test, which is a cycle ergometer test more specific to cycling-based sports. The development of the Repeated Ability Sprint Test (RAST) provides a reliable, valid (Zagatto et al., 2008), and practicable field test to determine running anaerobic power (Nick and Whyte, 1997). With 6 × 35 m repeated sprints, the total running time is close to 30 s, making the test comparable with the Wingate test. Times and body weight can be used to calculate maximal and average power outputs along with a fatigue index. Repeated high-intensity sprints cause substantial metabolic acidosis, contributing to muscular fatigue and metabolic output decline (Kairouz et al., 2013).

Therefore, the aims of this pilot study were to determine the feasibility of implementing the WHBM before sport performance and evaluate whether a single WHBM session provides any acute ergogenic effects during repeated-sprint bouts. In addition to performance, physiological and psychological data were collected to allow a better global understanding of the WHBM.

## Methods

### Experimental Approach to the Problem

We did a randomized, controlled three-way crossover pilot study to (1) assess the feasibility of pre-performance WHBM and (2) compare the acute effects of single sessions of the WHBM, HV, and SB on performance. Feasibility concerned recruitment, execution of the WHBM, and data collection. Performance was assessed with the RAST. Body mass and running times were used to calculate peak power, average power, and fatigue index (FI) (Nick and Whyte, 1997) to compare performance between conditions. Gas exchange, oxygen saturation (finger pulse oximetry), heart rate (HR), rate of perceived exertion (RPE), and responses to three questionnaires were collected to evaluate whether there were any differences in RAST performance between the breathing methods that would correlate with physiological changes.

### Participants

Inclusion criteria included being a healthy regular runner (in base training for more than 3 years with 2 or more running training sessions per week) in order to limit variability in sprinting performance, being familiar with maximal sprinting, being an adult (18 years or older), and being male (to avoid influence from hormone level changes and to limit the total number of participants to be recruited). The participants were recruited through word of mouth and social media. Sixteen physical education students volunteered with one participant excluded because of an injury, resulting in a final sample size of 15 (72.4 ± 6.3 kg, 24.5 ± 2.3 years, 10.3 ± 4.7 training years, 7.6 ± 2.5 h/week training, 7.3 ± 1.1 peak sprint power in watt/kg; mean values ± standard deviations).

### Procedures

The experiments were performed in late summer 2020 on a covered 50 m tartan track (“La Pontaise,” Lausanne, Switzerland, elevation 597 m). Weather influenced ambient temperature, which was systematically measured. Once a week (same day, same time) for 4 weeks; each participant was seen by the same experimenter. The first meeting was used to answer questions and obtain written consent, to record demographic data, to collect a baseline running time with the control condition, and to familiarize the participants with the testing and the two breathing maneuvers to maximize reliability. The next three sessions were used to assess each method (WHBM, HV, and SB) in a balanced randomized order (computer-generated sequence). The participants were kept blinded to the results of the sprints until the end of the last session. They were instructed to maintain their usual diet and lifestyle during the study, to abstain from tobacco, coffee, alcohol, and ergogenic drugs the day of testing, and to abstain from any important exercise the day leading up to the test.

Participants started each session with the same self-chosen warm-up. Then, they took a supine position on a floor mat and rested for 3 min 30 s for the acquisition of resting values before executing the specific breathing method (WHBM, HV, and SB) while conserving the supine position. Then, after 10 s, the participants prepared for the start of the RAST. The test involved performing 6 × 35 m sprints with 10 s rests between sprints. The participants took a sprint 3-point stance start position 3 s before each sprint, waited for the “go” signal, and then performed all-out sprints under strong verbal encouragement. The participants were instructed to perform each sprint as fast as possible and not use a pacing strategy. The timing was started manually and automatically stopped with photocells using an electronic timer (Witty, Microgate, Bolzano, Italy). Upon arrival, the participants immediately took their supine position again for 10 min of post-sprint measurements.

#### Breathing Methods

**Figure 1:**
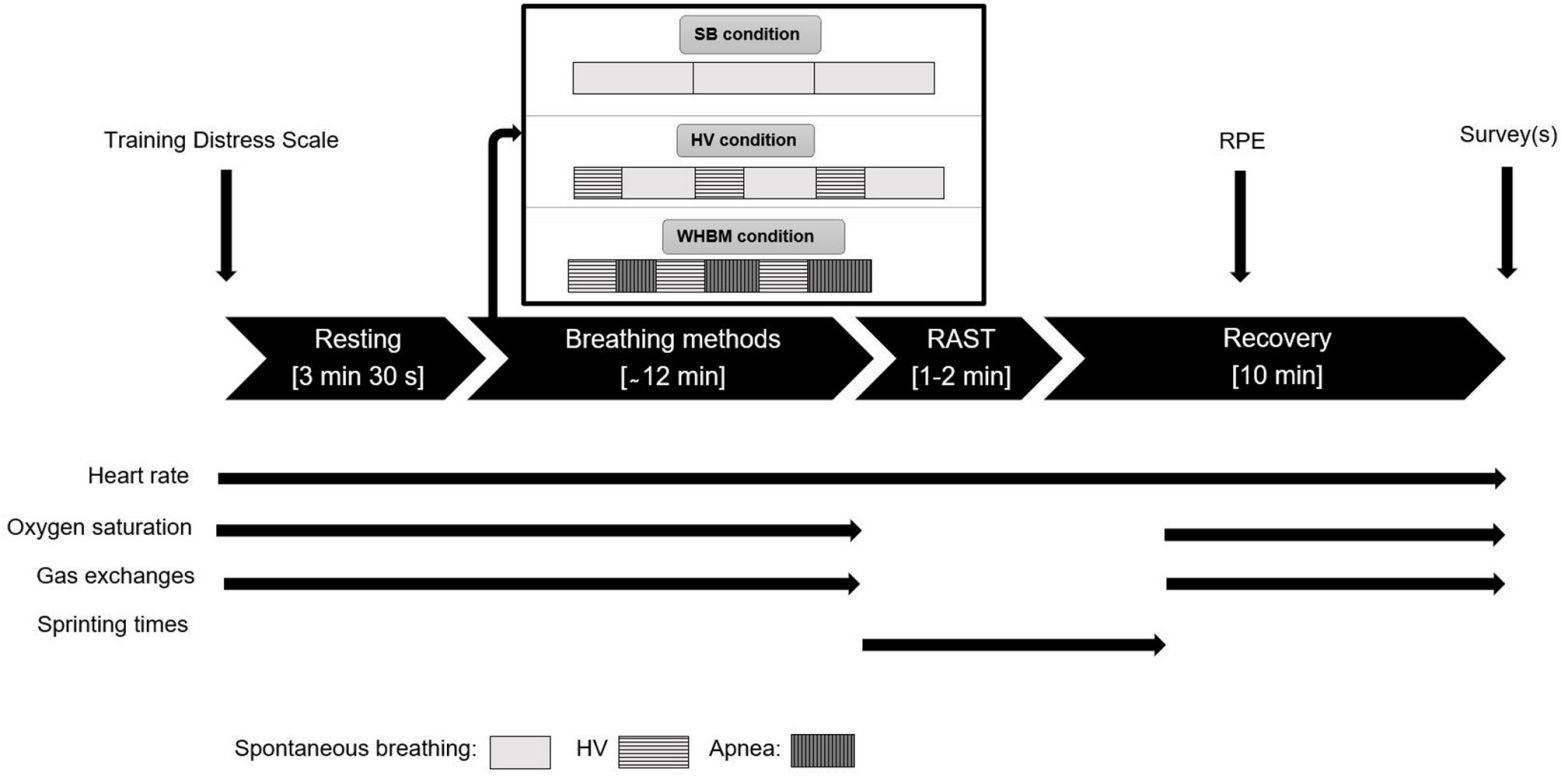
Overview of the protocol. Thin straight black arrows indicate when different data collection occurred. Apnea rectangles length increases because the breath-holds (BHs) durations spontaneously increased.

The WHBM was performed using the audio guide on the WHM mobile app. The participants performed three cycles of the WHBM as prescribed by the official website (Hof, 2020b). One cycle consisted of hyperventilating for 30 breaths, defined as respiratory movements of maximum amplitude at the frequency given by the audio guide (~0.32 Hz). This period lasted ~1 min 30 s. The participants then fully exhaled to residual volume (RV) and held their breath as long as possible (BH). At breaking point, they inhaled to total lung capacity (TLC) and kept their breath for 15 s before starting the next cycle. HV was performed as in the WHBM, except that the BH times were substituted with ~2 min 30 s of spontaneous breathing (the prior estimated breath hold duration), for a total duration of 12 min for the breathing maneuvers. The control condition consisted of spontaneous breathing (SB) for 12 min. Upon completion of the last cycle, the participants took off the face mask and the finger oximeter and prepared for RAST (see Figure 1).

#### Repeated Sprint Ability

Performance was assessed with the RAST. Peak power and average power were obtained using the equation *Power* = (*bodymass* × *distance*^2^)/*time*^3^ and the FI was calculated using the equation *FI* = (*Peak Power* − *Min Power*)/*Total Sprint Time* (Nick and Whyte, 1997).

#### Physiological Monitoring

Except during RAST, gas exchange was monitored breath-by-breath throughout the experiment with a portable metabolic device (K5, COSMED, Rome, Italy) and a face mask. The device was calibrated before each test using a 3 L syringe and gas mixtures of known concentration. Delay calibration and scrubber testing were done regularly. Oxygen uptake (VO_2_), expired carbon dioxide (VCO_2_), and minute ventilation (VE) were averaged for 1 min during rest, for the entire HVs durations, for 5 breaths immediately after RAST, and for 1 min after 8 min 30 s of post-RAST recovery. End-tidal O_2_ partial pressure (PETO_2_) and end-tidal CO_2_ partial pressure (PETCO_2_) were averaged for 1 min during rest, for the 5 last breaths during HV, and for the 5 last breaths pre-sprint. During the spontaneous breathing periods between HV, values were averaged for the entire duration. To obtain post-RAST cumulated oxygen uptake (VO_2_-OFF), cumulated expired carbon dioxide (VCO_2_-OFF), and cumulated ventilation (VE-OFF), breath-by-breath data were cumulated for 9 min post-sprint and the volumes equivalent to 9 min at rest (pre-RAST) were subtracted. PETCO_2_ values were used to estimate pH levels according to the algorithm of Siggaard-Andersen (Siggaard-Andersen and Siggaard-Andersen, 1990) and to estimate pH variations according to Dubose's equation (Dubose, 1983): pH variation = 0.08 × (40 – PETCO_2_ measured) / 10.

Apart from during RAST, middle finger pulse oxygen saturation (SpO_2_) was recorded throughout with an oxygen saturation monitor (Pulsox PO-400, Contech Medical Systems, Qinhuangdao, China). Data were averaged for 2 min during pre-RAST resting, for 5 s at approximately the highest value during hyperventilation, for 5 s at approximately the lowest value during breath holds, for the entire duration during the spontaneous breathing intervals in the HV condition, and for the 10 s pre-RAST.

HR was collected throughout with a thoracic belt and wristwatch (H10 belt and V800 watch, Polar Electro Oy, Kempele, Finland). HR data were filtered using custom routines (MATLAB, the Mathworks, Nattick, MA, USA) with detection and compensation of ectopic beats, median filtering to remove isolated outliers, detection of block errors, and replacement by interpolated values. Data were then averaged over 1 min during rest, over 5 beats around the lowest value during breath holds in WHBM; over 5 beats around the lowest value during spontaneous breathing periods between hyperventilation periods; over 5 beats around the highest value during hyperventilation; for 750 s, 30 s, 5 beats, for the SB, HV, and WHBM conditions, respectively, before RAST; over 5 beats for HR recovery after 1 min; and over 5 beats for HR recovery after 2 min. During RAST, the highest HR was retained as HRmax. HR reserve percentage was calculated using the Karvonen Formula: *HR reserve percentage* = (*HRmax measured during sprints* −*Resting HR*)/(*HRmax predicted* −*Resting HR*), where HRmax predicted was calculated as 220 - age.

#### Questionnaires

Prior to each session, the participants completed the Training Distress Scale as a performance readiness assessment (Grove et al., 2014). RPE was recorded on a Borg CR10 scale 1 min after the RAST. A custom questionnaire was used for the subjective assessment of the three sessions. The items were ≪ *I felt negative effect(s) of this way of breathing used before the repeated sprint test* ≫ : yes/no, and if yes, which one(s); ≪ *I felt positive effect(s) of this way of breathing before the repeated sprint test* ≫*:* yes/no, and if yes, which one(s); ≪ *To perform the test, this way of breathing made me feel overall* ≫*:* visual analog scale going from strongly disadvantaged to strongly advantaged; and ≪ *I plan to reuse this way of breathing in the future in my personal practice* ≫*:* yes/no.

Upon completion of the last session, we added the following additional items: ≪*Rank from 1 to 3 the methods you felt the best in preparation for sprinting (1 being the best and 3 being the worst)* ≫ and ≪*Rank from 1 to 3 the method you think you performed the best with (1 being the best and 3 being the worst)* ≫. Study data were collected and managed using REDCap electronic data capture tools hosted at UniSanté, Lausanne, Switzerland. An overview of the data collection is shown in Figure 1.

### Statistical Analysis

As no data were available for power calculations since this research was a pilot study, none were performed. For the resting physiological measurements, breathing method physiological measurements, RAST and recovery physiological measurements, and questionnaire results, Shapiro-Wilk's test was used to ensure variable normality. If normality was ensured, condition effects were analyzed using linear mixed models (participants as the random effect and condition as a fixed effect) in two steps. First, to exclude period and carry-over effects of the cross-over trial, we included the effect of time or sequence (fixed effects) as an addition to the model. When time and sequence had no effect, they were removed in the second step. There was a sequence effect for HR at pre-sprint and for the respiratory frequency at rest, and a time effect for VE at HV1. Thus, they were considered in the second step. Normality was not found for Fatigue Index (WHBM condition), for resting PETCO_2_ (WHBM condition), for resting VCO_2_ (SB and HV condition), for VCO_2_ at HV1 (SB condition), for VCO_2_ at HV2 (SB condition), for VCO_2_ at HV3 (SB and HV condition), for VCO_2_ post recovery (SB condition), for VCO_2_-OFF (SB condition), and for visual analogic scale ≪ *To perform the test, this way of breathing made me feel overall* ≫ (SB condition). A non-parametric repeated measures analysis (Friedman test) was performed for these variables. Statistical tests were corrected for multiple comparisons using Bonferroni correction. While these tests were performed using SPSS, version 26 (IBM Corp., Armonk, NY, USA), repeated measures correlation tests were performed between VCO_2_-OFF and subjective variables with rmcorr library of R version 4.0.5 (R Foundation for Statistical Computing, Vienna, Austria). For all tests, significance was set at *p* ≤ 0.05.

## Results

Checking of respect for the protocol, the temperature records, and the performance readiness survey results assured that the experiments occurred in reliable and valid conditions. Due to data recording errors, SpO_2_ was not saved for two participants in the WHBM condition and respiratory parameters were not saved for one participant in the SB condition.

### Feasibility and Performance

Out of 16 participants, 1 participant was excluded because of a muscular injury (in the lower limb during the first sprint), resulting in a final sample size of 15. The participants were able to perform the WHBM and HV methods without difficulties. Only minor malfunctions or technical problems occurred during data collection. As described in the section Surveys, 47% of the participants asserted to use the WHBM in the future while 53% did not. The values of RAST peak power, average power, and FI are shown in Table 1. There were no significant differences between conditions.

**Table 1:** Performances determined during the repeated ability sprint test (RAST) realized with SB, HV, and WHBM conditions.

	**SB**	**HV**	**WHBM**	***p***
Peak power (watts)	501.9 ± 104.5	492.3 ± 112.7	501.3 ± 115.6	0.720
Average power (watts)	418.2 ± 84.3	407.6 ± 85.8	413.6 ± 86.7	0.360
Fatigue index	4.6 ± 2.2	4.7 ± 2.2	4.9 ± 1.7	0.819

*SB, spontaneous breathing; HV, hyperventilation; WHBM, Wim Hof Breathing Method*.

*Mean values ± standard deviations*.

### Resting Physiological Measurements

Resting values are presented in Table 2. A significant difference was found in VE between SB and WHBM conditions (*p* = 0.039) and in VCO_2_ between HV and WHBM conditions. According to the algorithm of Siggaard-Andersen (Siggaard-Andersen and Siggaard-Andersen, 1990), PETCO_2_ values indicated resting pH values of 7.380, 7.397, 7.397 for the SB, HV, and WHBM conditions, respectively.

**Table 2:** Resting values in SB, HV, and WHBM conditions.

	**SB**	**HV**	**WHBM**	***p***
HR (bpm)	75 ± 10	77 ± 10	79 ± 12	0.122
SpO_2_ (%)	96 ± 1	96 ± 1	96 ± 1	0.298
PETO_2_ (mmHg)	102 ± 3	102 ± 5	103 ± 5	0.570
PETCO_2_ (mmHg)	42 ± 7	40 ± 3	40 ± 4	0.167
VO_2_ (ml/min)	366 ± 36	364 ± 70	400 ± 63	0.119
VCO_2_ (ml/min)	414 ± 54	403 ± 105	449 ± 90^‡^	**0.017**
VE (L/min)	11.2 ± 1.4	11.4 ± 2.8	12.7 ± 2.0^†^	**0.025**
Tidal volume (mL)	1068 ± 288	1123 ± 383	1174 ± 287	0.595
Respiratory frequency (/min)	12 ± 4	11 ± 3	12 ± 3	0.881

*SB, Spontaneous Breathing; HV, Hyperventilation; WHBM, Wim Hof breathing method*.

*Mean values ± standard deviations*.

† *Significantly greater than SB*.

‡ *Significantly greater than HV. Bold values indicate significant differences*.

### Breathing Method Physiological Measurements

Table 3 presents HR max, SpO_2_ max, PETO_2_ max, PETCO_2_ min, average VO_2_, average VCO_2_, average VE, average tidal volume, and average respiratory frequency during the hyperventilation periods for the WHBM condition. It further lists HR min, SpO_2_ min, and BH duration during the BH periods. For the HV condition, the same parameters during the hyperventilation periods are shown; HR min and averaged values for the other parameters during the spontaneous breathing periods between the hyperventilation periods are also shown. Pre-sprint values are presented for both conditions.

**Table 3:** Physiological measurements during WHBM and HV conditions.

		**HR** **(bpm)**	**SpO**_**2**_ **(%)**	**PETO**_**2**_ **(mmHg)**	**PETCO**_**2**_ **(mmHg)**	**VO**_**2**_ **(ml/min)**	**VCO**_**2**_ **(ml/min]**	**VE** **(L/min)**	**Tidal volume** **(ml)**	**Respiratory frequency** **(1/)**	**BH duration** **(s)**
WHBM condition	HV1	110 ± 13.2	99 ± 0	128 ± 2	22 ± 3	501 ± 79	1344 ± 213	61.9 ± 9.2	3148 ± 537	25 ± 19	–
	BH1	67 ± 15	77 ± 9	–	–	–	–	–	–	–	74 ± 16
	HV2	108 ± 14	99 ± 0	128 ± 2	20 ± 3	673 ± 128	1179 ± 181	61.2 ± 10.9	3135 ± 553	20 ± 2	–
	BH2	67 ± 1	68 ± 10	–	–	–	–	–	–	–	101 ± 19
	HV3	108 ± 16	99 ± 1	128 ± 2	19 ± 3	730 ± 129	1112 ± 201	61.4 ± 13.3	3112 ± 773	22 ± 8	–
	BH3	66 ± 14	60 ± 12	–	–	–	–	–	–	–	119 + 26
	Pre-sprint	80 ± 17	62 ± 12	–	–	–	–	–	–	–	–
		^Ø^	^¥^		^‡^						^¥^
HV condition	HV1	104 ± 12	99 ± 1	128 ± 2	21 ± 3	488 ± 89	1308 ± 208	59.5 ± 12.8	2979 ± 672	22 ± 7	–
	SB1	71 ± 12	97 ± 1	108 ± 6	30 ± 2	299 ± 68	310 ± 83	11.6 ± 3.3	1065 ± 368	12 ± 3	–
	HV2	104 ± 13	99 ± 1	129 ± 2	18 ± 2	511 ± 124	1136 ± 201	61.6 ±14.0	3072 ± 859	23 ± 13	–
	SB2	68 ± 9	98 ± 1	107 ± 7	26 ± 3	292 ± 75	249 ± 87	10.8 ± 4.2	988 ± 381	12 ± 3	–
	HV3	102 ± 12	99 ± 0	129 ± 2	17 ± 3	542 ± 120	1030 ± 161	60.9 ± 13.0	3130 ± 696	20 ± 4	–
	SB3	66 ± 11	98 ± 1	109 ± 8	24 ± 3	329 ± 82	266 ± 95	12.8 ± 5.4	1144 ± 480	13 ± 4	–
	Pre-sprint	72 ± 13	97 ± 2	105 ± 10	25 ± 4	411 ± 111	294 ± 116	13.5 ± 5.7	1076 ± 559	15 ± 6	–
				^#^	^†^						

*SB, spontaneous breathing; HV, hyperventilation; WHBM, Wim Hof breathing method*.

*Mean values ± standard deviations*.

*–, Non available data*.

Ø *Indicates that, in average, HR significantly increased with the HVs and significantly decreased with the BHs compared to resting values in WHBM condition*.

¥ *Indicates that values were significantly different between BH1, BH2 and BH3 in WHBM condition*.

# *Indicates that values were significantly greater of resting value in HV1, HV2 and HV3 in HV condition*.

† *Indicates that pre-sprint value was significantly greater than HV3 in HV condition*.

‡ *Indicates that values were significantly different between HV1 and HV2, and between HV1 and HV3 in WHBM condition*.

**Figure 2:**
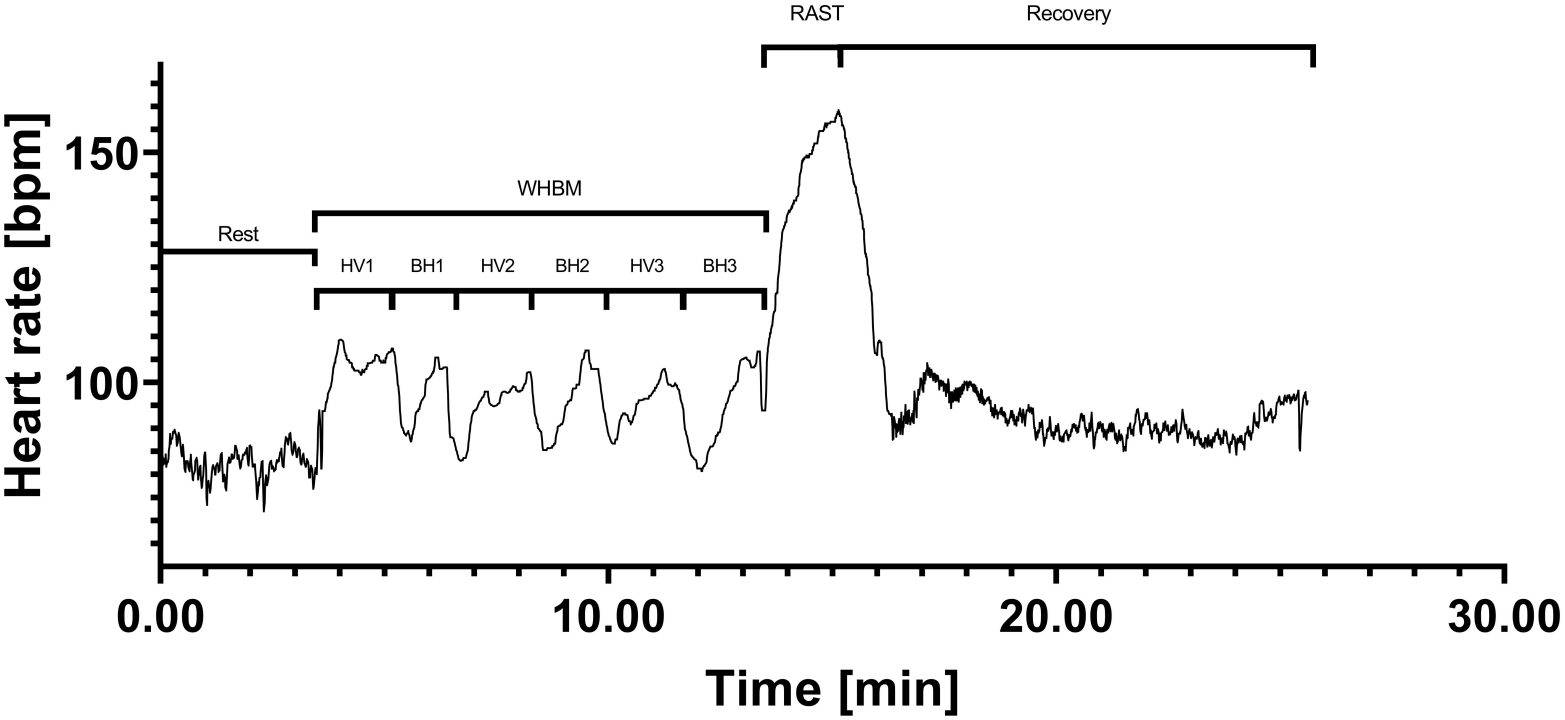
Participant 7 heart rate (HR) data in the Wim Hof breathing method (WHBM) condition.

**Figure 3:**
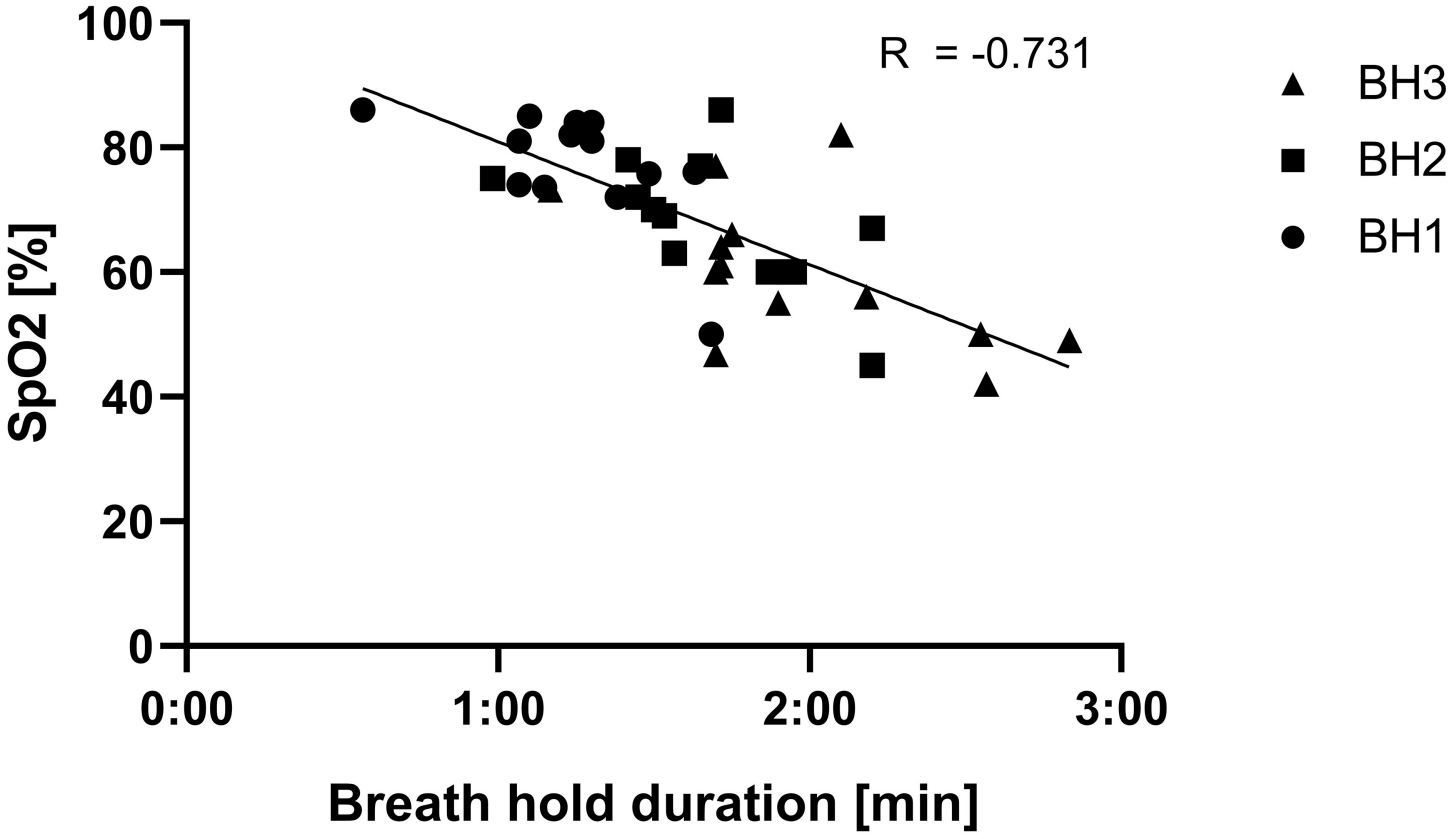
Relation between breath hold durations following hyperventilations (HVs) and oxygen saturation (SpO2) in the Wim Hof breathing method (WHBM) condition.

The single inhalation to TLC following breath holding at RV to breaking point generally induced a sharp increase of HR. HR then decreased again during the 15 s additional BH at TLC. An example of a HR pattern is presented in Figure 2. A strong correlation (*r* = −0.731) between BH duration and SpO_2_ was found (Figure 3). In the WHBM condition, VO_2_ during HV2 and HV3 was −288 ± 182 ml and −437 ± 207 ml, respectively, inferior to assumed VO_2_ consumption during the respective previous BH (resting VO_2_ × BH duration). Assuming no net change in blood buffer status, according to the algorithm of Siggaard-Andersen (Siggaard-Andersen and Siggaard-Andersen, 1990), the minimal PETCO_2_ values reached at the end of HV3 corresponded to a pH of 7.651 in the WHBM condition and 7.688 in the HV condition, confirming a state of respiratory alkalosis. The pre-RAST value for the HV condition corresponded to a pH 7.559, suggesting partial correction of the respiratory alkalosis. Similarly, according to Dubose's equation (Dubose, 1983), the minimal PETCO_2_ reached at the end of HV3 corresponded to a pH variation of +0.171 units pH in the WHBM condition and +0.181 in the HV condition. The pre-RAST value for the HV condition corresponded to an increase of +0.123 units pH.

**Figure 4:**
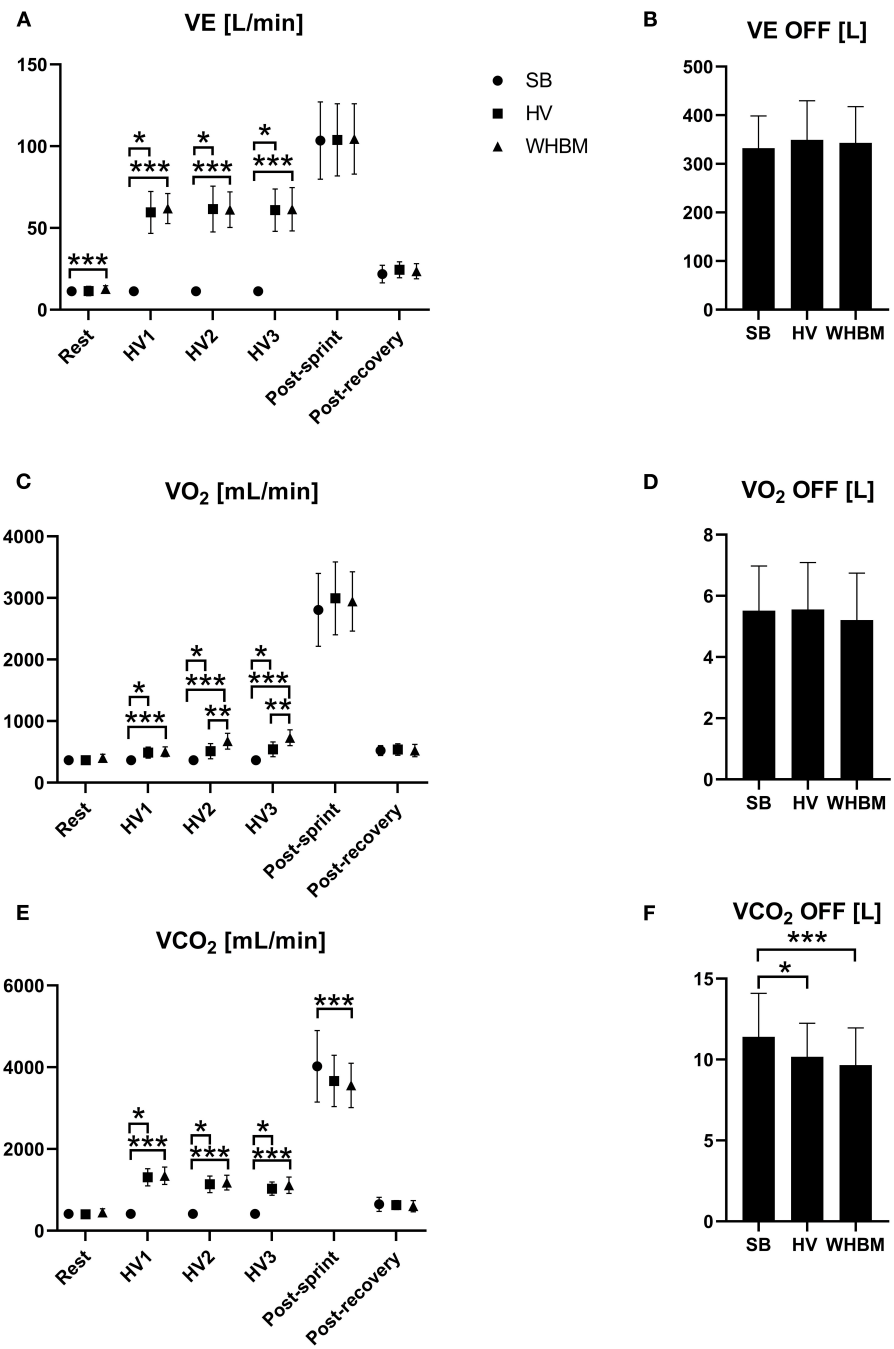
**(A)** Changes in minute ventilation (VE), **(B)** cumulated ventilation (VE-OFF), **(C)** oxygen uptake (VO_2_), **(D)** cumulated oxygen uptake (VO_2_-OFF), **(E)** expired carbon dioxide (VCO_2_), and **(F)** cumulated expired carbon dioxide (VCO_2_-OFF) for SB, HV and WHBM conditions. SB, spontaneous breathing; HV, hyperventilation; WHBM, Wim Hof Breathing Method. ^*^indicates a significant difference between SB and HV condition, ^**^indicates significant difference between HV and WHBM condition, ^***^indicates significant difference between SB and WHBM condition.

The average values of VE, VO_2_, and VCO_2_ measured at rest, during HV1, HV2, and HV3, immediately upon arrival after RAST, and during recovery in all three conditions are shown in Figure 4.

### RAST and Recovery Physiological Measurements

Table 4 presents RAST and recovery heart rate. No significant differences were found between the conditions for any of these measurements.

**Table 4:** Repeated ability sprint test (RAST) and recovery heart rate during WHBM and HV conditions.

	**SB**	**HV**	**WHBM**	***p***
Pre-sprint (bpm)	76 ± 8	72 ± 13	80 ± 17	0.083
Highest HR during sprint (bpm)	173 ± 8	172 ± 7	173 ± 8	0.122
Highest percentage of HR reserve during sprints (%)	81 ± 7	80 ± 7	80 ± 7	0.063
HR recovery after 1 min (bpm)	–49 ± 14	–48 ± 10	–48 ± 13	0.755
HR recovery after 2 min (bpm)	–62 ± 9	–62 ± 9	–61 ± 10	0.477

*SB, spontaneous breathing; HV, hyperventilation; WHBM, Wim Hof breathing method*.

*Mean values ± standard deviations. HR recovery values indicate post exercise drops in HR from highest HR during sprint, in bpm*.

The values of VE, VO_2_, and VCO_2_ measured post-RAST and post-recovery are shown in Figure 4 with VE-OFF, VO_2_-OFF, and VCO_2_-OFF.

### Surveys

RAST Borg CR10 scores were 7.5 ± 1.2, 7.6 ± 1.1, and 6.9 ± 1.4 in SB, HV, and WHBM conditions, respectively. Statistical differences were found for WHBM condition versus HV (*p* = 0.008) and versus SB condition (*p* = 0.017).

Negative effects of tingling, numbness, dizziness, and heaviness were reported by 60% of the participants for the HV condition. Negative effects of heaviness and deafness were reported by 33% of participants for the WHBM. Positive effects of improved breathing and less fatigue were reported by 73% of the participants for the HV condition. Positive effects of improved breathing, less fatigue, and increased energy were reported by 87% of the participants for the WHBM condition. Finally, 47% reported planning to reuse the WHBM in their personal training and competitive practice in the future while 53% declared not to.

**Figure 5:**
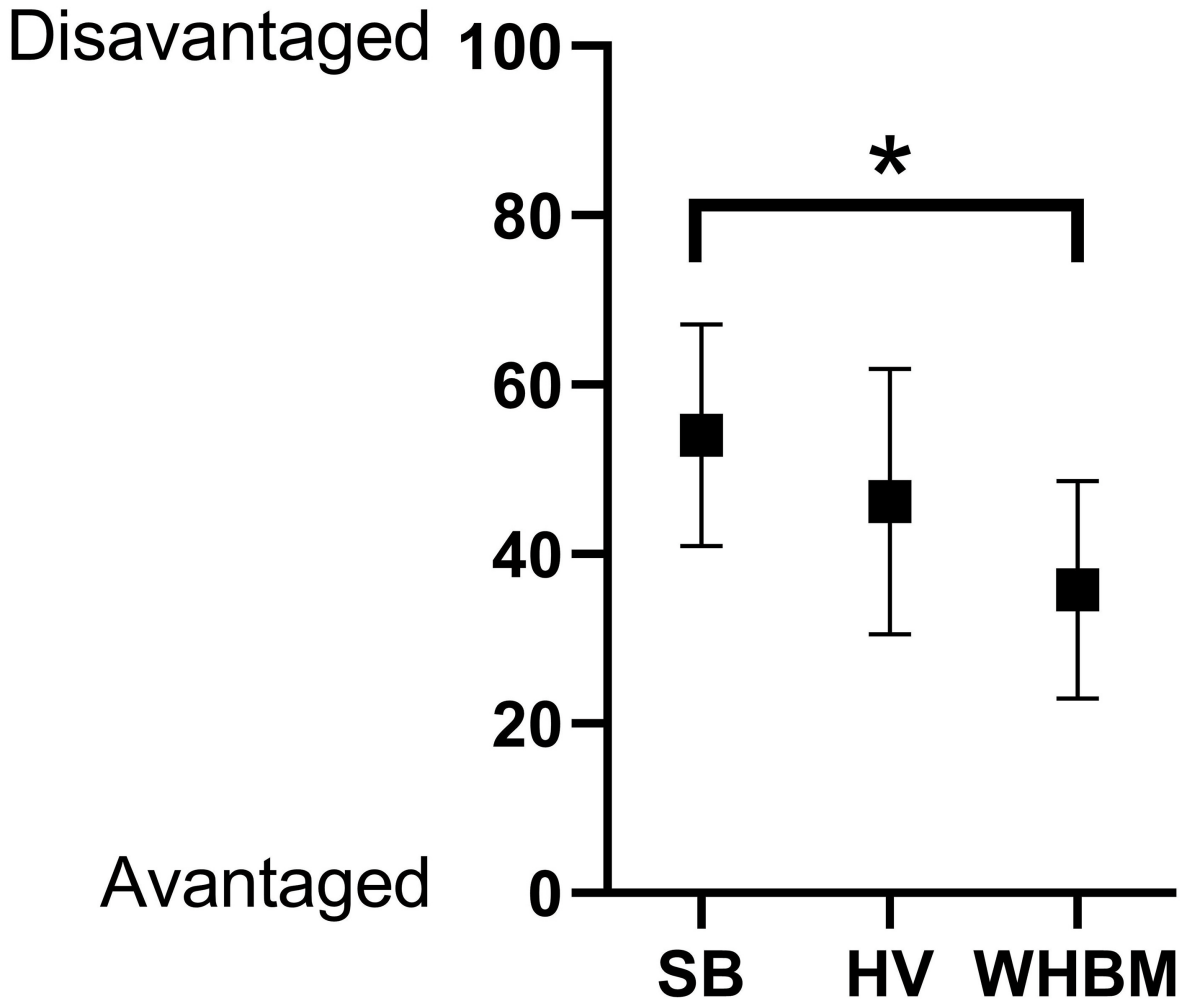
Results to visual analogic scale “To perform the test, this way of breathing made me feel overall” (mean ± standard deviation). ^*^p < 0.05.

Participants felt the most advantaged when performing the test in the WHBM condition, followed by the HV condition (Figure 5). A significant difference was found between WHBM and SB conditions.

Overall assessment of the breathing methods provided the results shown in Table 5. A majority of participants assessed WHBM as the best and SB as the worst.

**Table 5:** Overall assessment of breathing methods in percentage of participants.

		**SB**	**HV**	**WHBM**
The best / the worst method in term of sprint test	The best	13.3%	13.3%	73.3%
	The worst	53.3%	46.7%	0.0%
The best / the worst method in term of perceived performance	The best	13.3%	20.0%	66.7%
	The worst	53.3%	46.7%	0.0%

## Discussion

### Feasibility and Performance

This study reports the feasibility and effects of practicing the WHBM before repeated sprinting performance. The acute effects of a single session of WHBM were assessed on repeated-sprint bouts and on various physiological and psychological variables. After receiving anecdotal information from early adopters, we expected that the use of WHBM might give an edge for sprinting performance. Participants consented to comply and adequately performed the WHBM and procedures, contributing to a valid assessment. Despite some lightheadedness and tingling, they were able to perform the RAST as required. However, in spite of large physiological effects of both HV and WHBM, no significant condition effect was found regarding performance, peak power, average power, or fatigue index. Apart from the CO_2_ stores depletion that persisted through the RAST (VCO_2_-OFF), the observed physiological effects were specific and immediate to the respiratory maneuvers, and they did not translate into global physiological repercussions that could change performance. It follows that, despite subjective preference for the two breathing methods in comparison with spontaneous breathing, in the present experimental conditions, the acute application of two specific breathing methods did not convey any advantage for repeated sprinting performance as assessed with the RAST. However, the lower post-sprint CO_2_ levels in HV and WHBM conditions could be a factor that explains the subjective preference. Indeed, VCO_2_ levels are linked to respiratory distress in dyspnea and apnea studies. Although weak, a correlation between VCO_2_-OFF and the survey item ≪ *Rank from 1 to 3 the method you think you performed the best with* ≫ was found (*r* = −0.445, *p* = 0.014). However, the subjective preference could also reflect some placebo mechanism.

### Respiratory Alkalosis

PETCO_2_ values indicated a blood pH increase of +0.171 (to reach an estimated pH of 7.651) upon the last HV in the WHBM condition, which could have had ergogenic effects on anaerobic performance by preventing and/or compensating exercise-induced metabolic acidosis (Jacob et al., 2008). However, in the WHBM condition, the HV-induced respiratory alkalosis was attenuated by the ensuing BH-induced CO_2_ retention. Indeed, PETCO_2_ values decreased along the HVs to a lower level in HV compared to the WHBM condition (down to 17 ± 3 versus 19 ± 3 mmHg, respectively). This difference was not significant, but it can be hypothesized that it increased slightly with the last BH. Additionally, HVs effects were probably also attenuated during the delay before the RAST. In the HV condition, PETCO_2_ rose pre-sprint to values that corresponded to a +0.123 pH change (and an estimated pH of 7.564) instead of +0.181 (and a pH of 7.682)] at the end of the last HV. Thus, we speculate that the WHBM pH change was slightly inferior to +0.123 before beginning the RAST. In agreement, another study on WHBM reported that a representative subject ended the last BH with a pH of 7.50, a value 0.10 higher than at the start (Kox et al., 2014). In comparison, other studies reported improved performance using HV with comparable or smaller pH changes (Sakamoto et al., 2014; Jacob et al., 2015), greater pH changes (Ziegler, 2002), and smaller pH changes using bicarbonate supplementation (Costill et al., 1984; Bishop et al., 2004). However, other studies did not find improved performance even though comparable or smaller pH changes were reported (Jacob et al., 2008; Kairouz et al., 2013; Sakamoto et al., 2018). While the literature is not unanimous, HV, by inducing respiratory alkalosis, may have positive effects on anaerobic type performance.

### Diving Response

BH by itself triggers the so-called diving response, and when coupled with stimulation of facial cold receptors, a greater response is seen (Foster and Sheel, 2005). The diving response includes bradycardia, peripherical vasoconstriction, increased blood pressure, and contraction of the spleen (Dujic et al., 2011). Breath-holding is known to induce spleen contraction leading to an increase of blood hemoglobin concentration (Schagatay et al., 2001). Spleen contraction releases stored erythrocytes into the circulation. A single contraction causes a hemoglobin increase that corresponds to a 3–10% increase in blood oxygen carrying capacity (Stewart and McKenzie, 2002). The increased hemoglobin levels may have potentially beneficial effects on performance for both increased blood O_2_ carrying capacity and increased CO_2_ buffering capacity (Schagatay et al., 2012). However, the increased blood pressure and peripheral vasoconstriction might impair performance. Studies evaluating the effect of apneas on performance found no improvement (Du Bois et al., 2014; Sperlich et al., 2015; Yildiz, 2018). In consistence with these studies, the WHBM did not enhance performance in this investigation.

### Blood Oxygen Saturation

Oxygen saturation dropped to very low levels at the end of the BHs (60%). The 10 s delay before the start of RAST might not have allowed full recovery of saturation, which could have impaired performance. In another study assessing an apnea test following a forced expiration, oximetry recovery times were between 20 and 40 s (Plas and Bourdinaud, 1953).

### Catecholamine and Cortisol Levels

In another investigation (Kox et al., 2014), the authors reported that, while WHBM led to no increase in norepinephrine, dopamine, and cortisol levels, significantly higher plasma epinephrine levels were found. The latter has a powerful vasodilator effect on blood vessels in skeletal muscle (Davis et al., 2008) and stimulates glycogenolysis (Kenney, 2012), which could lead to ergogenic effects in the WHBM condition.

### Gas Exchange

Comparable VE was observed during HVs in the HV and WHBM conditions. Compared to spontaneous breathing, VO_2_ showed significantly higher values for the HV condition, which is possibly linked to greater work performed by the respiratory muscles (Coast et al., 1993). VE was further increased in the WHBM condition, likely due to the BH-induced oxygen desaturation leading to a hypoxic ventilatory response (Figure 4C). Intriguingly, the difference in VO_2_ consumption between the WHBM and HV conditions during HV2 and HV3 was inferior to assumed VO_2_ consumption during the previous BH. VCO_2_ showed significantly higher values in the HV condition as a result of larger CO_2_ elimination, and it rose to an even greater extent in the WHBM condition given the CO_2_ accumulation during the post-HV BHs. However, this difference was not significant (Figure 4E). VCO_2_-post after RAST in the WHBM condition was significantly smaller than in the SB condition, while in the HV condition it was not, which is counter-intuitive given the CO_2_ accumulation during BHs. In addition, it is interesting to note that VCO_2_-OFF was significantly smaller in both the WHBM and HV conditions than in the SB condition, which suggests that some of the HV-induced CO_2_ stores depletion had persisted through the RAST.

### Breath-Hold Duration

BH durations were comparable to the durations most people can achieve with no prior HV and at full lung volume. We speculate that several factors counterbalanced each other, resulting in these durations. The most potent regulator of ventilatory drive is pH, followed by partial pressure of carbon dioxide (PCO_2_), and, to a lesser extent, partial pressure of oxygen (PO_2_) (Saladin and Miller, 2004). There is also a substantial prolongation of BH time that is independent of chemical stimuli, which has been attributed to neural input from pulmonary stretch receptors (Mithoefer et al., 1953). Thus, apnea at low lung volume as performed in WHBM leads to shorter BHs because of the decreased stimulus to the pulmonary stretch receptors and accelerated onset of hypoxia and hypercapnia (decreased O_2_ pulmonary volume and decreased capability to dilute the rise in metabolically-derived CO_2_ levels) (Skow et al., 2015). However, considering that HV reduces arterial CO_2_ content and increases arterial pH, the subsequent BH duration should be longer, as it will take longer to reach the threshold of chemoreceptor activation (Skow et al., 2015). Consistent with previous findings (Schagatay et al., 1999), the latter is also the more likely reason as to why BH duration significantly increased between sets: the successive HVs progressively reduced arterial PCO_2_ as suggested by the decreasing PETCO_2_ values from one to the other HV. Under such conditions, arterial PO_2_ can decrease to a greater extent (Djarova et al., 1986) and trigger spleen contraction as described above. In this study, oxygen saturation dropped progressively through the BH sets and reached severe hypoxemia levels (60%) at the end of the last BH, which is consistent with another WHBM investigation where values reportedly even decreased to about 50% (Kox et al., 2014). The correlation between SpO_2_ and BH duration (Figure 2) further illustrates the above explanations. In BHs following HV, activation of the peripheral chemoreceptors from sensing increased CO_2_ is delayed, and their activation from sensing decreased O_2_ is increased. If SpO_2_ decreased to a great extent during BHs, it resumed normal values during the HVs, i.e., almost fully saturated (Kenney et al., 2012). Consistent with these studies, PETO_2_ significantly increased above resting value during HV1, HV2, and HV3 in this study, indicating the effect of hyperventilation on alveolar gas composition.

### Heart Rate

In the WHBM condition, HR significantly increased (by 31 bpm on average) during the HVs and significantly decreased (by 13 bpm on average) during the BHs compared to resting values. The HR increases during HVs were probably due to increased motor drive for increased respiratory muscle activity (Cummin et al., 1986). The HR drops during the BHs are generally attributed to an increase in cardiac parasympathetic drive triggered in response to breathing cessation (Cherouveim et al., 2013). However, the HR drops were also observed in the HV condition; thus, HV cessation may be the main explanation. The sharp HR increase following the first inhalation after BHs observed in this study has been previously described. Upon resumption of breathing after an apnea test following forced expiration, pulses give way sometimes to an irregular acceleration, which tends to stabilize quickly (10–15 s) at approximately its initial rate (Plas and Bourdinaud, 1953). Because of these mechanisms, pre-sprint HR was not significantly different between conditions. During the sprints, HR reserve percentage reached testified to the high intensity of the effort.

### Surveys

Psychological assessment showed rather positive results in the WHBM condition; RPE was significantly the lowest, participants felt significantly advantaged the most to perform the test, and felt the best for doing the sprint test and thought they performed the best. Around three-quarters of them reported positive effects such as less fatigue, increased energy, and improved breathing. Speculatively, the latter could potentially be due to reduced work of breathing resulting from pre-activation of sympathetic drive and catecholamine secretion, leading to bronchodilation and decreased airflow resistance (Sakamoto et al., 2014). On the other hand, negative effects such as deafness or dizziness were reported, potentially caused by the HV induced hypocapnia causing cerebral vasoconstriction (Skow et al., 2015). In the end, around half of our participants declared that they would consider the WHBM in their personal practice in the future. The participants reported significantly lower RPE with the WHBM. This may be due to a lesser “maximality” of the sprinting effort or reflect some placebo mechanism. The participants were necessarily aware that the experiment aimed to assess the effects of two breathing methods on repeated sprinting performance. Their belief that the WHBM might be beneficial could have led to such results or even worse, it may even have negated a decreased performance from WHBM-induced changes in physiological variables such as SpO_2_.

### Limitations

Several limitations should be considered when interpreting the results of this research. First, the relatively small sample size affected the reliability of the study. Second, because one is supposed to do a deep inspiration from RV to TLC 15 s before the end of the BH in WHBM and arterial blood gas analysis was not feasible, it was not possible to document pulmonary or blood gas exchanges during and at the end of the WHBM BHs. Also, there are several studies in the literature expressing reservations about the reliability of SpO_2_ in situations leading to deep hypoxemia (Pottecher et al., 2003), indicating that the SpO_2_ results during the BHs should be interpreted with caution. Third, fatigue index values were rather low compared to other studies: 7.1 in students of physical education and sport exercise (Paradisis et al., 2005), 5.4 after a power endurance training in basketball players (Balčiunas et al., 2006), 8.1–10.5 in elite basketball players (Pojskić et al., 2015), 4.2 in football players, 4.2 in sprinters, 4.2 in takrawer, 2 in volleyballers, and 4.6 in Pencak Silater athletes (Nasuka et al., 2019). The results could have been different if the participants had reached higher FI. A sensitivity analysis in this study on the four participants with a FI superior to five did not show statistical differences either. Finally, the participants described starting the RAST after resting motionless for ~12 min in a supine position as difficult. Vigorous respiratory muscle contraction in the WHBM and HV conditions and maximal apnea in the WHBM condition could have mitigated these negative effects.

Another investigation could evaluate using the WHBM sitting on a chair or standing to limit the difficulty previously mentioned even if it would involve a less optimal breathing position. It would also be interesting to assess effects of apnea followed by HV, as potential ergogenic apnea-induced spleen contraction could last long enough and potential ergogenic HV-induced effects would be optimized if exercise was performed without delay. Moreover, while this study focused on acute effects of the WHBM, it would be of interest to assess its regular use in combination with training. Repeated-sprint training in hypoxia has been shown to induce greater improvement of repeated-sprint performance than in normoxia (Brocherie et al., 2017). Also, repeated sprint-induced arterial desaturation through voluntary hypoventilation at low lung volume induced greater enhancement in competitive swimmers than in normoxia (Trincat et al., 2017), and the magnitude of the improvement (+35%) was comparable to that obtained with repeated sprinting in hypoxia in cycling (+38%) (Faiss et al., 2013) and in double poling cross-country skiing (+58%) (Faiss et al., 2015). Similar or even greater improvements might be expected with a regular use of the WHBM.

## Practical Applications

This investigation is the first to evaluate the WHBM in view of improving repeated sprinting performance. While this pilot study underlined the possibility of practicing an acute session of WHBM before sport performance, it also presented side effects and did not enhance any performance parameter (peak power, average power, and FI) in later sprint sets. It appears unworthy to carry this method out as the improved physiological parameters did not translate into a performance increase. Based on the results found in this study, we do not recommend applying this method with the view of improving performance, at least not for repeated sprinting.

## Data Availability Statement

The raw data supporting the conclusions of this article will be made available by the authors, without undue reservation.

## Ethics Statement

The studies involving human participants were reviewed and approved by Research Ethics Committee of the Canton Vaud. The patients/participants provided their written informed consent to participate in this study.

## Author Contributions

KG, BK, FC, and TC: conception and design, analysis and interpretation of data, drafting the article, critically revising the article, and final approval of the article. TC and KG: acquisition of data. All authors contributed to the article and approved the submitted version.

## Conflict of Interest

The authors declare that the research was conducted in the absence of any commercial or financial relationships that could be construed as a potential conflict of interest.

## Publisher's Note

All claims expressed in this article are solely those of the authors and do not necessarily represent those of their affiliated organizations, or those of the publisher, the editors and the reviewers. Any product that may be evaluated in this article, or claim that may be made by its manufacturer, is not guaranteed or endorsed by the publisher.

## Abbreviations

BH: breath-hold
FI: fatigue index
HV: hyperventilation
RAST: repeated ability sprint test
RPE: rate of perceived exertion
RV: residual volume
SB: spontaneous breathing
TLC: total lung capacity
WHBM: Wim Hof breathing method
WHM: Wim Hof method.
